# Membrane-less microfiltration using inertial microfluidics

**DOI:** 10.1038/srep11018

**Published:** 2015-07-08

**Authors:** Majid Ebrahimi Warkiani, Andy Kah Ping Tay, Guofeng Guan, Jongyoon Han

**Affiliations:** 1 School of Mechanical and Manufacturing Engineering, Australian Centre for NanoMedicine, University of New South Wales, Sydney, NSW 2052, Australia; 2 BioSystems and Micromechanics (BioSyM) IRG, Singapore-MIT Alliance for Research and Technology (SMART) Centre, Singapore; 3 Department of Biomedical Engineering, National University of Singapore, 117575, Singapore; 4 Department of Electrical Engineering and Computer Science, Department of Biological Engineering, Massachusetts Institute of Technology, Cambridge, Massachusetts, USA

## Abstract

Microfiltration is a ubiquitous and often crucial part of many industrial processes, including biopharmaceutical manufacturing. Yet, all existing filtration systems suffer from the issue of membrane clogging, which fundamentally limits the efficiency and reliability of the filtration process. Herein, we report the development of a membrane-less microfiltration system by massively parallelizing inertial microfluidics to achieve a macroscopic volume processing rates (~ 500 mL/min). We demonstrated the systems engineered for CHO (10–20 μm) and yeast (3–5 μm) cells filtration, which are two main cell types used for large-scale bioreactors. Our proposed system can replace existing filtration membrane and provide passive (no external force fields), continuous filtration, thus eliminating the need for membrane replacement. This platform has the desirable combinations of high throughput, low-cost, and scalability, making it compatible for a myriad of microfiltration applications and industrial purposes.

Microfluidics technology, introduced about two decades ago, has facilitated new progress in chemistry, biology, engineering, and medicine^1^,^2^. With channel dimensions matching typical cell sizes, microfluidics is poised to contribute significantly to cell biology^3^, for example, by providing more accurate control and manipulation than any conventional techniques. Yet, micro-scale manipulation naturally meant a small fluid volume processing rate, which is acceptable in analytical chemistry but not in many industrial processes, where economy of scale is important. Recent developments in inertial microfluidics^4^,^5^,^6^ and other high throughput microfluidic systems, therefore, are especially exciting since they have the potential to enable various microfluidic applications^7^ in those large scale industrial processes.

In order to showcase the potential of such ‘macro-microfluidics’, we developed a membrane-less microfiltration platform for ultra-high throughput (up to 500 mL/min) cell separation with extremely high yield using inertial microfluidics. Our system is a highly multiplexed microfluidic device consisting of multiple Polydimethylsiloxane (PDMS) layers with embossed microchannels (i.e., ~200 individual spiral microchannels) engineered for a continuous size-based sorting of cells from large volume of biological fluid. Individual separation channels are connected internally and biological sample fluid enters via a shared inlet, and exit through two outlets. Inside the curvilinear microchannels, cells, subject to hydrodynamic forces, display preferential migration to either outlet. Filtration and fractionation can therefore occur on the same platform, dependent on the magnitude of the net hydrodynamic forces. The utility of this system were demonstrated by carrying out large-scale mammalian cell retention from bioreactors (i.e., flow rate of ~500 mL/min), yeast cell separation, and cell synchronization. As cells are separated solely due to hydrodynamic forces driven by externally-driven flow, our system can run continuously, without the need for membrane filter replacement that consume the majority of operating cost of any filtration system.

## Working principle

Neutrally buoyant particles (or cells) suspended in a fluid flowing through a straight microchannel experience a net inertial lift force arising from the balance between shear induced and wall induced lift forces^8^,^9^. By adding curvilinearity to the channel design, two-counter rotating vortices in the top and bottom half of the channel (i.e., Dean vortices) will be formed, which apply a drag force on the particles (*F*_*D*_). The force balance between *F*_*L*_ and *F*_*D*_ determines the equilibrium positions of the particles in curvilinear channels^10^.

As both forces are a function of particle size (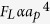
and 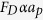)^8^, particles of different sizes occupy distinct lateral positions near the channel wall and exhibit different degrees of focusing, allowing size-based separation. Additionally, the inertial lift force is a function of Reynolds’ number (Re) and decreases with increasing Re^8^. Drag vortices can also be understood using the Dean number which is a linear function of Re^6^. As Re changes, there are opposite effects on the magnitude on the inertial lift forces and Dean drag. The balance between the two forces therefore leads to particle equilibrium.

**Figure 1:**
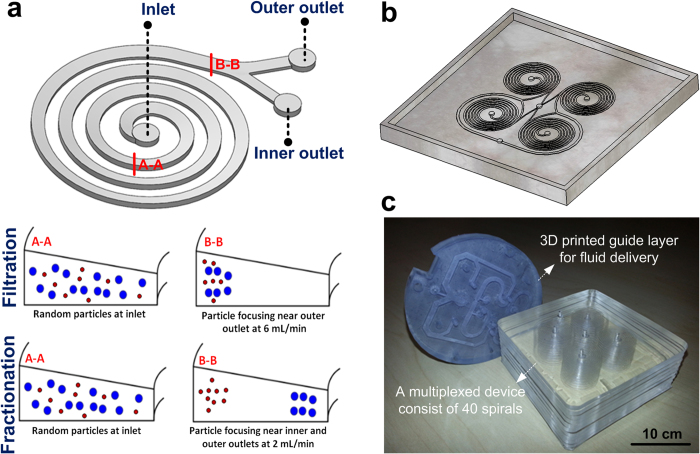
(**a**) Schematic of a trapezoidal cross-section spiral microchannel illustrating the principle of particle focusing and trapping within the Dean vortices. In the filtration mode, all the suspended particles inside the fluid are trapped and focused near the outer wall where strong vortices exist. In the fractionation mode, smaller particles are trapped inside the Dean vortices and remained near the outer wall while bigger particles are focused near the inner wall, thus allowing particle separation at the outlets. The key to changing from one mode to another relies on the magnitude of the hydrodynamic forces in the microchannels that are in turn relies on particle sizes, flow rates and channel dimensions. (**b**) Schematic 3D drawing of a high-throughput module consists of four spirals connected together. This design is employed to create the master mold using precision micromilling, and then used in soft lithography to replicate PDMS layers. (**c**) Optical image of a high-throughput system consists of multiple PDMS layers with embossed microchannels (i.e., 40 multiplexed devices) bonded for continuous cell retention from large sample volumes along with a 3D printed guide layer for fluid delivery. The throughput of this system (a single unit) can be as high as 240 mL/min or ~350 L/Day. This throughput can be further increased (up to thousand litres per day) through parallelization.

Recently, we have shown that by altering channel cross-section from rectangular to trapezoidal, we can create stronger Dean vortex cores near the outer wall for trapping smaller particles thus enhancing the separation throughput and efficiency^11^,^12^. Spirals with trapezoidal cross-section are able to function effectively in both the filtration and fractionation mode. The majority of the suspended particles can be trapped near the outer wall by strong vortices at a certain flow rate, hence facilitating filtration. Additionally, by optimising the channel dimensions to particle size ratio and flow rates, smaller particles can be trapped near the outer wall while larger particles focused near the inner wall, enabling smooth fractionation (Fig. 1).

## Results

To demonstrate the suitability of our system for large-volume applications, we have used our system for three distinct microfiltration purposes i.e., cell retention from perfusion bioreactors, yeast filtration, and cell cycle synchronization.

### Membrane-less cell retention from bioreactors (CHO and yeast cells)

Mammalian cells are the expression systems of choice in the pharmaceutical industry due to their ability to synthesize large and complex proteins for biotechnological and medicinal purposes^13^. Yeasts are eukaryotic microorganisms that play valuable roles in industrial processes such as alcohol production^14^. Currently, large-scale cell cultures in bioreactors are typically performed in stirred, fed-batch or perfusion modes^15^,^16^. Both volumetric production and product concentration in perfusion cultures can be much higher than in a fed-batch type cultures^17^, due to continual feeding of nutrients and removal of waste^18^. Yet, implementation of perfusion cultures has been impeded by the challenge of long-term, large-scale cell retention in bioreactors^19^. Among various techniques (Table S1), membrane microfiltration and centrifugation are the two most commonly used for cell retention^20^. Large scale centrifuges are capital and energy intensive equipment^21^, and the use of centrifugation also requires batch processing, limiting the outputs of production. In microfiltration, membrane fouling and clogging is the most significant issue that plagues virtually all filtration membranes^22^. Despite the various improvements made over the last several decades (*e.g.* cross-flow filtration) for fouling mitigation during or after filtration, membrane fouling and clogging still remain as the major challenge for filtration.

**Figure 2:**
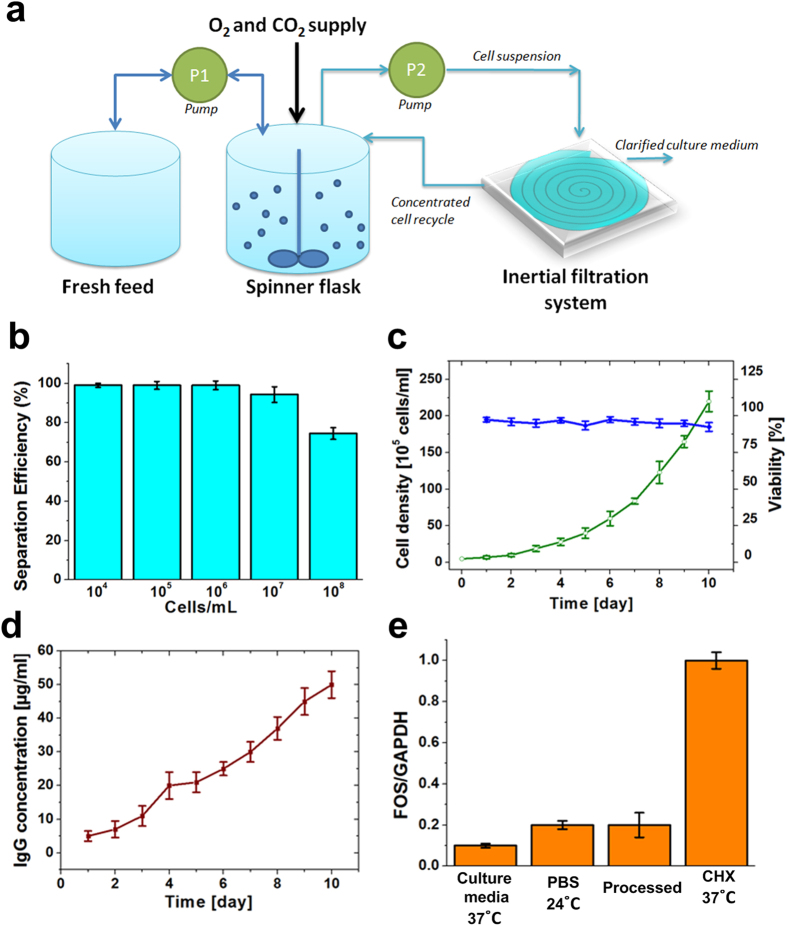
(**a**) Sample processing workflow showing process of cell enrichment using the high throughput filtration system from spinner flasks imitating condition of a perfusion bioreactor. Cell cultures are subject to inertial filtration system and used media are assessed for product e.g., IgG concentration. Fresh media and enriched cell samples are added back to the flask following each filtration process. (**b**) Separation efficiency of CHO cells as a function of concentration at 6 mL/min flow rate for a single spiral device. (**c**) Viable cell density and viability of hybridoma cells for 10 days continuous culture. The results indicated that both parameters are not significantly affected by inertial filtration. (**d**) Rate of IgG production by hybridoma cells over 10 days. A steady increase in IgG was observed, suggesting that cellular activities involved in IgG production were minimally affected by inertial filtration (**e**) Evaluation of stress levels of processed cells compared to cells incubated at 37 °C in cell culture media, or at 24 °C in the PBS buffer. Up-regulation of *c-FOS* gene was evident in response to cycloheximide (CHX) treatment^38^ which was considered a positive control. The gene expression was normalized to the GAPDH housekeeping gene for CHO cells for different conditions.

To demonstrate the feasibility of our system as a clog-free cell microfiltration system, cell cultures were performed using 250 mL disposable spinner flasks inside a humidified incubator. Microfiltration tests were conducted daily by isolating the used media from cell cultures using our inertial filtration system in a sterile environment while fresh media was replenished to each flask along with enriched cells following each experiment (Fig. 2a). Cell densities, viability, glucose, antibody titers and pH were monitored in each sample separately. Microfiltration tests revealed utility of our system for continuous cell separation from bioreactors over a range of concentrations, with over 95% efficiency at flow rate of 6 mL/min for a single spiral. 6 mL/min flow rate was optimized after testing a flow rate from 1–10 mL/min for a single chip. To demonstrate the ease of multiplexing to achieve desired flow rate in our technology, 84 spiral chips are multiplexed to provide a combined flow rate of 500 mL/min instead. Due to the relatively large channel designs (~mm range) in our inertial microfiltration system, cell concentrations up to 10^7^ cells/mL can be accommodated in our system with negligible effect on separation efficiency; however, for concentrations higher than this value, the separation efficiency decreased (Fig. 2b). These results have been confirmed for three different cells lines (Fig. S1). The viability of the sorted cells was similar to that of the unsorted (control), with more than 90% of the cells excluding the propidium iodide dye, suggesting minimum cellular damage during separation (Fig. 2c). These results were further confirmed by seeding a fraction of sorted cells for proliferation and growth observations. The morphologies and proliferation rate of the isolated CHO cells was similar to the control cells with no noticeable differences (Fig. S2). Our group also showed that inertial separation of mesenchymal stromal cells (MSCs) did not induce any detectable changes in their viability and differentiation potential into different lineages^23^. These results support that our developed inertial filtration technique has minimal effect on the cells during isolation while maintaining high post-sorting cell viability (also see video S1 &S2). Cell productivity was also assessed by measuring activity of the secreted IgG protein using an enzymatic assay (Fig. 2d). Our results demonstrated sustainable growth of the cells and antibody production over a period of 10 days, suggesting the value of this new technology for separation of animal cells from the culture medium. The expression profile of c-*FOS* gene was investigated as an indicator of shear in our system (Fig. 2e). The *c-FOS* is involved in the regulation of early signal transduction pathways by modulating expression of multiple genes as a part of the AP-1 transcriptional activator complex and the shear stress inducibility of *c-FOS* protein has been shown in human and animal cells lines of different origins^24^,^25^. Expression of this gene was evaluated on CHO cells and obtained results showed that fluidic shear did not induce up-regulation of *c-FOS* gene as compared with the two negative controls: cells incubated in culture media at 37 °C and cells in the PBS buffer at 22 °C. This can be attributed to the low residence times of cells in the microfluidic channels (<0.1 sec).

### Yeast filtration

**Figure 3:**
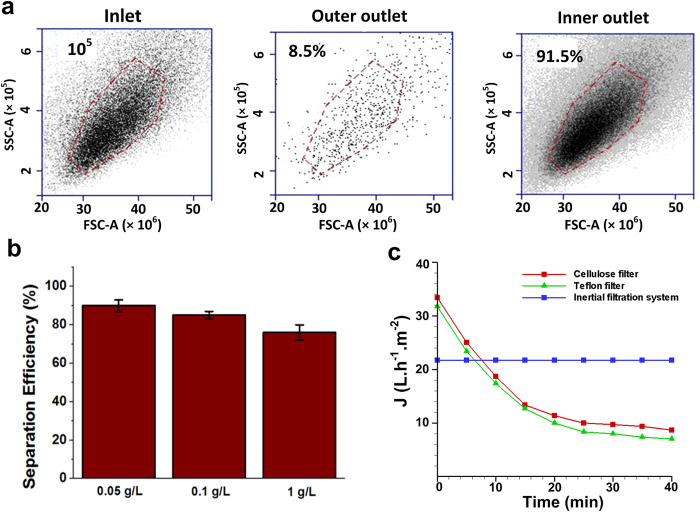
(**a**) Scatter plots captured using a flow cytometer showing the effectiveness of inertial microfiltration systems for enrichment of small microorganism such as yeast at 0.05 g/L concentration and flow rate of 2 mL/min (for a single spiral). More than 90% separation efficiency can be achieved using this system through a single cycle processing. (**b**) Separation efficiency of yeast cells as a function of concentration at 2 mL/min flow rate for a single spiral device. Samples with high yeast concentration (1 g/L) can be also processed using inertial system with relatively high efficiency. The data also confirms the suitability of our filtration system for a wide range of cells with different sizes as long as channel dimensions and flow rates are optimized. (**c**) The flux of two commercial microfilters and inertial microfiltration system for filtration of yeast at 0.1 g/L concentration. During the filtration runs, the flux of membrane filters gradually decreased due to the deposition of yeast on the membrane surface and fouling; however, our inertial system without any physical barrier presented superior performance in terms of throughput during the course of filtration. The inertial microfiltration unit used for comparison purpose comprised of 180 spirals channels working at flow rate of around 320 mL/min.

To demonstrate another application of our high-throughput filtration system, *Saccharomyces cerevisiae* was used as a model of small microorganism (~3–5 μm) to test the cell sorting capacities of our membrane-less microfiltration platform. The small size of yeasts mandates the modification of channels dimensions to focus them near the side wall at certain flow rates. For this particular application, trapezoidal channel with 30 × 70 μm dimensions is found to optimise yeast cell isolation. Accordingly, a new mold accommodating eight small spirals in a single layer was employed as a base platform for multiplexing our system to achieve higher throughputs (Fig. S3). Rehydrated yeast suspension was created by diluting a dry strain of *S. cerevisiae* (Sigma-Aldrich) in water into a concentration of 0.1 g/L. Microfiltration tests using yeast cells at optimised flow rate of 2 mL/min for a single spiral (i.e., ~320 mL/min for the multiplexed system) supported the feasibility of inertial microfluidics for high-throughput yeast separation for broth fermentation (see video S3). The separation of yeast cells at concentration of 1 × 10^5^ cells/mL showed over 90% separation efficiency (Fig. 3a).

Importantly, at higher concentrations of yeast suspension, our device demonstrated a reasonable performance with just one cycle of separation (Fig. 3b). The separation efficiency can be further enhanced by increasing the number of sorting cycles. To evaluate the suitability of our system for industrial purpose, comparisons were made between our system and two widely used commercial membrane filters namely, Cellulose acetate and Teflon filters for filtration of yeast. Microfiltration results using these three systems made it evident that our inertial microfluidic system outperformed the membrane-based system in long term sample processing mainly due to its continuous, low-cost and membrane-less operation (Fig. 3c).

### Cell cycle synchronization

Using the similar system, we also demonstrated cell synchronization of large volume cell culture. The ability to effectively isolate highly synchronized populations of cells has several biotechnological utility. For instance, developments of anti-cancer drugs can be expedited with the use of synchronized cell populations as these drugs typically target specific cell cycle phase to limit cancer proliferation^26^. Also, synchronizing cells such as Chinese Hamster Cells (CHO)-derived cell line that grow more rapidly can boost production of products such as recombinant proteins^27^,^28^.

Existing methods to synchronize cell populations, such as using chemical agents (e.g. Nocodazole)^29^, serum starvation^30^ and contact inhibition, can compromise cell viability, induce apoptosis and affect metabolism in subsequent cell generations^31^. Another way to obtain synchronized cell populations is using counterflow centrifugal elutriation (CCE)^32^, but such a technique reportedly exerts unnecessary stresses on the cells, leading to undesirable biophysical and biochemical alterations^33^.

**Figure 4:**
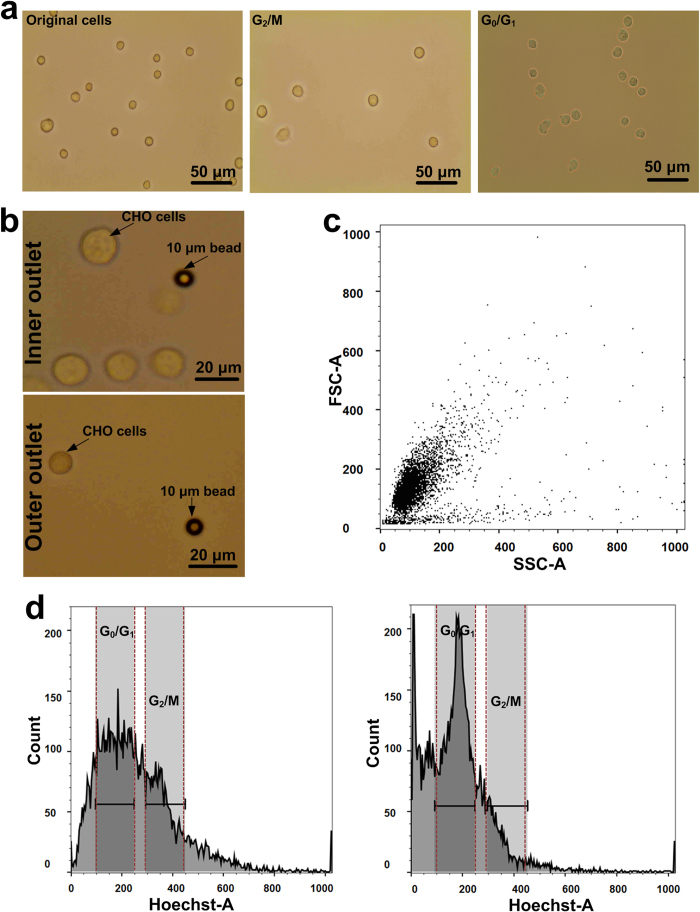
Results demonstrating the effectiveness of the inertial filtration system for cell synchronization. (**a**) Left to right: Optical image of original cell population before sorting. CHO cells present displayed a range of sizes i.e. 11–20 μm. Optical image showing CHO cells arrested in G_2_/M phase with Nocodazole. Cell sizes were 18 ± 2 μm. Optical image showing CHO cells arrested in G_0_/G_1_ phase via contact inhibition. Cell sizes were 11 ± 2 μm. (**b**) Top & bottom: optical image showing CHO cells in the inner and outer outlets respectively after separation with their sizes compared to 10 μm beads at 1.5 mL/min. The cells at the inner outlet have larger sizes while that in the outer outlet have smaller sizes. (**c**) The positive slope of the forward and side scatter graphs indicated the correlation between cell size and cell-cycle phase. (**d**) *Left*: histogram of the DNA content showed that before sorting, the G_0_/G_1_:G_2_/M ratio was 1.82:1. Distinct peaks could be observed for cells in the G_0_/G_1_ and G_2_/M phase by their DNA content stained with Hoechst. *Right:* after sorting at 1.5 mL/min (1 × 10^6^ cells/mL), the G_0_/G_1_:G_2_/M ratio improved close to three folds to 5.02:1 in the outer outlet. The G_0_/G_1_ purity in the outer outlet was ~ 80%, an improvement from ~ 60% in the unsorted sample. The increase in cell count in the outer outlet also indicated selective enrichment of cell population with lower DNA content in the G_1_ phase. This can be further improved by processing output from the inner outlet again (2^nd^ or 3^rd^ cycles).

Previously, our group made use of inertial forces in spiral microchannel (i.e., with rectangular cross-section) to isolate an array of cells such as CHO and marrow-derived human mesenchymal cells (hMSCs)^34^. In this study, we demonstrate another application of our novel high-throughput system for large-scale cell synchronization suitable for industrial applications. In the fractionation mode (see Fig. 1a), cells typically ≥14 μm are isolated via the inner outlet while smaller cells, which are trapped in the strong vortices near outer wall, exit the spiral device via the outer outlet. Capitalizing on the size differences between cells in G_0_/G_1_ and G_2_/M phase (with the latter being larger, Fig. 4a,c), a single spiral microfluidic device is able to perform cell synchronization with a throughput of 1–2 mL/min (up to 1 × 10^6^ cells/mL) while the multiplexed system presented here can deliver throughput of 150–300 mL/min or even higher (see video S4). This throughput is high enough to fractionate multiple cell culture flasks or a perfusion reactor (i.e., 1–5 L capacity) within few minutes for downstream characterization of cells or re-inoculation of the system.

Similar to the previous sections, the separation performance of the device was first characterized with a binary mixture of latex particles (10&15 μm). Then, the synchronization performance of the CHO cells was measured such that the smaller (G_0_/G_1_) cells were enriched through the outer outlet while bigger cells (G_2_/M) were isolated via inner outlet (Fig. 4b). After the synchronization, the cells were fixed and stained with Hoechst for quantitative determination of the cell-cycle phase using FACS. In the initial asynchronous mixture, the ratio of the cells in G_0_/G_1_ phase to those in G2/M phase is 1.82:1. However, this ratio improved close to three folds to 5.02:1 after one round of sorting as confirmed by DNA staining via Hoechst which is a DNA intercalating dye (Fig. 4d).

Although this result is currently less than the four folds enrichment reported by other groups, it can be improved with increased rounds of sorting. Sorting also increased the G_0_/G_1_ cell population from ~60% to 80%, a feat comparable to reported literature. Taking into considerations the technical equivalence in terms of cell population purity and enrichment ratio and also the superior throughput of our spiral microfluidic device, we believe that our platform can be suitably adapted for large-scale industrial fractionation of cells.

## Discussion

The relatively low throughput of conventional microfluidic systems has been the principal impediment for industrial adoption of the technology in spite of many unique advantages of microfluidics. In this work, we show that this limitation can clearly be lifted, by demonstrating a highly multiplexed microfluidic platform capable of processing macroscopic scale sample volumes.

The separation efficiency of the inertial microfiltration system remains above 90% for various cell lines at different concentration during 10 days processing. The viability of all cell lines was not affected markedly by the applied shear stresses attributed to short residence time of cells inside the inertial filtration system. Both post-sorted and unprocessed hybridoma cells proliferated at similar rate with no significant decline in their production titer. These findings support that high-throughput inertial sorting does not perturb cellular homeostasis in stress-mediated pathways, probably because the acceleration of cells in this system is modest (~a few hundred *g*), compared with centrifugation techniques. Given the flexibility and relative ease of implementation of inertial microfluidics for passive sorting, we believe that this method of cell retention can be adopted easily in desktop perfusion bioreactors for continuous manufacturing of monoclonal antibodies.

Demonstration of continuous membrane-less filtration for yeast cells is especially meaningful, since microfiltration becomes progressively challenging as the particle / cell sizes decreases below 10 μm. Previously, an inertial focusing of particles as small as 0.78 μm has been experimentally demonstrated^35^, and this idea may be extended to nanoscale particles by scaling down the dimensions of the channels (nanofluidics). Therefore, continuous, membrane-less microfiltration using inertial microfluidics has the potential to disrupt the current membrane-based microfiltration technology, which is widely used not only in biomedical / pharmaceutical industry but also in fields such as desalination and water purification, food processing, mining, and many other industrial separation processes that relies on membrane filtration.

## Methods

### Device design and fabrication

The high-throughput inertial filtration system was fabricated in polydimethylsiloxane (PDMS) using standard microfabrication soft-lithographic techniques described previously^36^. The master mold with specific channel dimensions was designed using SolidWorks^®^ software and then fabricated by conventional micro-milling technique (Whits Technologies, Singapore) on aluminium for subsequent PDMS casting. The spiral device used in this study for mammalian cell retention and fractionation was an 8-loop spiral microchannel with one inlet and two outlets with radius increasing from 8 mm to 24 mm for efficient cell migration and focusing. Four of these spiral channels were connected together systematically in one layer of PDMS, and the final multiplexed system was created by bonding multiple layers of PDMS using plasma treatment (Harrick Plasma, USA) and manual alignment. The PDMS layers were fabricated by casting degassed PDMS (mixed in a 10:1 ratio of base and curing agent, Sylgard 184, Dow Corning Inc.) on the mold and subsequent baking inside an oven for 2 hours at 70 °C. The fluidic access holes were punched inside the device using Uni-Core™ Puncher (Sigma-Aldrich Co. LLC. SG) and the final device was irreversibly bonded to a thick layer of plain PDMS using an oxygen plasma machine to complete the channels. The assembled device was finally placed inside an oven at 70 °C for 2 days to further enhance the bonding.

For efficient fluid delivery, we have used 3D printing (SLA stereolithography system, USA) to create a guide layer with internal fluidic channels and precise conical pins, which can be inserted into the filtration system and distribute the fluid equally in all the microchannels.

The optimized width and height for CHO cells separation are 600 μm and 80 × 130 μm while that for yeast cells are 450 μm and 30 × 70 μm.

### Cell culture

The AE-1 hybridoma cell line was purchased from the American Type Culture Collection (ATCC Cat. No. HB-72) cells were cultured in Dulbecco’s Modification of Eagle’s Medium (DMEM) (Invitrogen, USA) supplemented with 10% fetal bovine serum (FBS) (Invitrogen, USA). Frozen vial cells were thawed and placed into pre-warmed media together with 1% penicillin/streptomycin (Invitrogen, USA). Viable cell concentration was determined via trypan blue (and propidium iodide) exclusion and dilutions made to seed a T75 flask at a concentration of 1 × 10^5^ viable cells/mL. Cultures were incubated at 37 ^o^C in a humidified atmosphere containing 5% (v/v) CO_2_. Cell from plastic flasks started from thaw (above) were seeded into the 125 and 250 mL disposable spinner flasks at 2 × 10^5^ of pre-warmed complete Hybridoma-SFM media (Gibco^®^, USA). Spinner flask cultures were placed on a magnetic stir plate set to 100 rpm. Daily media samples were assessed for cell viability, nutrient and metabolite analysis. Media samples were also collected from spinner flasks for protein production (IgG1) which was assessed by following the IgG1 Elisa protocol provided by Abcam (Cat. No. ab100548). The adherent CHO-K1 cells (ATCC Cat. No. CCL-61) and MDA-MB-231 cell line were also cultured in low-glucose DMEM (Invitrogen, USA) supplemented with 10% FBS (Invitrogen, USA) together with 1% penicillin/streptomycin (Invitrogen, USA). The culture was maintained at 37 ^o^C in a humidified atmosphere containing 5% (v/v) CO_2_. The cells were sub-cultivated every 4 days with media replaced every 48 hours. Sub-confluent monolayers were dissociated using 0.01% trypsin and 5.3 mM ethylenediaminetetraacetic acid (EDTA) solution (Lonza, Switzerland). Glucose and lactate concentrations were determined using commercial assay kits from Sigma-Aldrich.

### Sample preparation

To characterize the performance of a single spiral device to build the multiplexed system based on that, we used fluorescent particles of varying size (10 and 15 μm to mimic the mammalian cells and 4 μm to mimic the yeast cells) as a surrogate for design optimization. Fluorescently labeled microbeads (Fluoresbrite^®^ Microspheres, Polysciences Inc, Singapore) were added (0.01% volume fraction) to sample buffer which consists of 1×phosphate buffered saline (PBS), 2 mM EDTA supplemented with 0.5% bovine serum albumin (BSA) (MiltenyiBiotec, Germany). BSA was used to prevent non-specific adsorption to the tubing and microchannel walls. The device was mounted (i.e., a single spiral device) on an inverted phase contrast/epifluorescence microscope (Olympus IX81, Olympus Inc., USA) equipped with a 12-bit EMCCD camera (iXon^EM^ +885, Andor Technology, USA). Samples were loaded within a syringe and pumped through the microchannel at varying flow rates using a syringe pump (Harvard Apparatus PHD 2000, Harvard Apparatus Inc., USA). Images were acquired using Metamorph^®^ software (Molecular Devices, USA) and analyzed using ImageJ® software. For tests with cell lines, high speed videos were captured at the channel outlet using Phantom Camera Control software (Vision Research Inc., USA) and then analyzed using ImageJ® software. Figure S4 presents superimposed fluorescent images of the microbeads captured at the outlet of a trapezoidal spiral microchannel (130 × 80 μm) at different flow rate. We have deigned our system in a way that at low flow rates (~1.5–2 mL/min); small particles in range of 10–14 μm get focused near the outer wall while bigger particles (>14 μm) get focused near the inner wall thus facilitating the fractionation. At high flow rates (~6 mL/min) sample processing, all the particles inside the microchannel can get focused near the outer wall, enabling filtration.

### Cell retention and fractionation experiments

For sorting experiments, cultured cells in spinner flasks (AE-1 hybridoma cell line) were filtered daily using our multiplexed inertial filtration system (i.e., we normally used a small version of our system consisting of 12–20 spirals working at flow rate of 60–100 mL/min) inside a sterilized environment while fresh media was added to each flask along with enriched cells during each experiments. Microfiltration tests were performed using a peristaltic pump (Masterflex, Cole-Parmer, USA) with silicon tubing and careful sterilization before and after each experiment. The flow ratio (output ratio of the two outlets) of our filtration system was set at around 50%. For adherent cells, subconfluent monolayers were dissociated with 0.01% trypsin and 5.3 mM EDTA and resuspended in the proper pre-warmed media for separation experiments. In the fractionation tests using CHO cells, the asynchronous cells were diluted to 1 × 10^6^ cells per mL in buffer containing 1x phosphate buffered saline (PBS), 2 mM EDTA supplemented with 1% BSA (Miltenyi Biotec, Germany) to prevent agglomeration and adsorption to the microchannel walls.

Although it is known that rehydrated yeast is not a perfect resemble of fresh cultivated cells, we have used dehydrated yeast in our tests for simplicity sake. This choice is also aligned with the industrial preference for dry yeasts over cultured yeasts nowadays. Rehydrated yeast also showed an average size of 3–5 μm that is similar to sizes of cultured yeast. The yeast suspensions were obtained from a dry strain of *Saccharomyces cerevisiae* (Sigma-Aldrich, USA) rehydrated in water at 35 °C for 10 min with stirring. After the rehydration, the yeast suspension was centrifuged for 15 min at 2000 g using a centrifuge and washed twice with distilled water to eliminate cell debris and soluble components. Then, the concentrated pellet was resuspended in the running buffer (explained above) and processed using the spiral system as well commercial microfilters. The microfiltration experiments using commercial membranes were performed in cross-flow mode using an in house module made from PMMA (Polymethyl methacrylate) by laser cutting and thermal bonding technique. The effective membrane area and height of the crossflow channel were 6.25 × 10^−4^ m^2^ and 0.0015 m, respectively (Fig. S5).

### Flow cytometry analysis

To arrest the cells by contact inhibition, the CHO cells were cultured in Rosewell Park Memorial Institute (RPMI) (Sigma-Aldrich, USA) for 96 h till 95% confluency. For M arrest by Nocodazole, CHO cells were cultured in RPMI with 10% FBS with 0.2 g/ml Nocodazole for 18 h. All cells were imaged without fixation. Flow cytometry analysis was conducted on the sorted samples to analyze the cellular DNA content via Hoechst. The sorted, synchronized cell populations were rinsed in 1x PBS and fixed in 4% (w/v) paraformaldehyde (PFA) for 15 min at 4 ^o^C. Cells were then centrifuged at 600 g for 5 min and incubated for 30 min in the staining solution containing 1x PBS, 3.8 mM sodium citrate (Sigma Aldrich, USA), 10 μg/mL RNase (i-DNA Biotechnology, Singapore) and Hoechst (NucBlue® LiveReadyProbes® Reagent, Invitrogen, USA). The stained cells were then tested for synchronization efficiency by performing flow cytometry (BD^TM^ LSR II flow cytometer, BD Biosciences, USA), and gated data were analyzed via FlowJo (FlowJo Ltd, UK) software.

### Gene expression analysis

For gene expression studies, CHO cells were pelleted and resuspended at a concentration of 1 × 10^6^ cells/mL in fresh media and PBS before processing using inertial filtration system. As a positive control, a fraction of cells were treated with 15 μg/mL cycloheximide (Sigma-Aldrich) at 37 °C inside incubator^25^,^37^. After processing through spiral microchannel, RNA was extracted immediately using the RNeasy Mini Kit (Qiagen) and transcribed into cDNA using the SuperScript III First-Strand Synthesis Kit (Invitrogen) according to the manufacturer’s instructions. The PCR primers as well as TaqMan probes for identification of C-FOS (# Hs00170630_m1) gene were purchased from LifeTech. GAPDH housekeeping gene was used for normalization of gene expression. The PCR Master Mix contained 1x primer/probe mix, 1x Universal Master Mix, and 500 ng of cDNA reaction in total volume of 25 μl. PCR amplification was performed using Bio-Rad PCR system at 50 °C for 2 min, 95 °C for 10 min followed by 45 cycles at 95 °C for 15 sec and 60 °C for 1 min. All the experiments were done in triplicate for RT-PCR determination of gene expression.

## Additional Information

**How to cite this article**: Warkiani, M. E. *et al.* Membrane-less microfiltration using inertial microfluidics. *Sci. Rep.* **5**, 11018; doi: 10.1038/srep11018 (2015).

## Supplementary Material

Supplementary Information

Supplementary Video S1

Supplementary Video S2

Supplementary Video S3

Supplementary Video S4
