# Supplementary material for: Membrane-less microfiltration using inertial microfluidics
Source: Sci Rep. 2015 Jul 8;5:11018. doi: 10.1038/srep11018 (PMC4495597; doi:10.1038/srep11018)
Supplement: Supplementary Information [file srep11018-s1.doc]

Membrane-less microfiltration using inertial microfluidics

**Majid Ebrahimi Warkiani, Andy Tay, Guofeng Guan, and Jongyoon Han**

**Supplementary information**

Table S1. Head to head comparison of inertial filtration system and existing techniques available in the industry for cell retention from perfusion bioreactors.

| **Technique/**  **Criteria** | **Filtration** | **Centrifugation** | **Hydrocyclones** | **Gravity sedimentation** | **Ultrasound/**  **electrophoresis** | **Inertial filtration system** |
| --- | --- | --- | --- | --- | --- | --- |
| **Cell viability (%)** | 50-90 | 70-85 | 80-85 | 88-100 | 70-95 | 95 |
| **Throughput^*^** | Medium | High | High | Low | Low | High |
| **Running cost** | High | High | Low | Low | Very High | Low |
| **Scalability** | Good | Good | Fair | Poor | Poor | Good |
| **Separation efficiency (%)** | 63-95 | 95-100 | >85 | >85 | >95 | >95 |
| **Cell Concentration**  **(10^6^ cells /mL)** | Typically  3-30 | 3-17 | 3 | 3-15 | 20-50 | Up to 50 |
| **Advantages** | Applicable to all cell types | High separation efficiency | Low cost, continuous processing | Low cost, high cell viability | High separation efficiency | Low cost, Continuous processing, High separation efficiency |
| **Disadvantages** | Low viability,  clogging | High capital cost | Not applicable to smaller cells | Too slow | Low throughput, High capital cost | -  -  - |

*High: tens to hundreds of mL/min range, Medium: hundreds of µL/min to mL/min range, low: tens of µL/min range.

**Table S2**. Reynolds’ number calculation for different trapezoidal channels used in this study.

|  | **Hydraulic diameter/ µm** | **Kinematic viscosity (of water/PBS)/ m^2^/s** | **Flow Velocity (×10^-9^ m^3^/s)** | **Channel Reynolds’ Number** |
| --- | --- | --- | --- | --- |
| **CHO cells (Bioreactor; channel dimensions: 80×130 µm)** | 89.84 | 1×10^-6^ | 100.2  (=6 mL/min) | 283.751 |
| **CHO cells (Cell cycle synchronization; channel dimensions: 80×130 µm)** | 178.460 | 1×10^-6^ | 16.7  (=1 mL/min) | 47.292 |
| **Yeast cells (channel dimensions: 30×70 µm)** | 178.460 | 1×10^-6^ | 33.4  (=2 mL/min) | 133.323 |

| **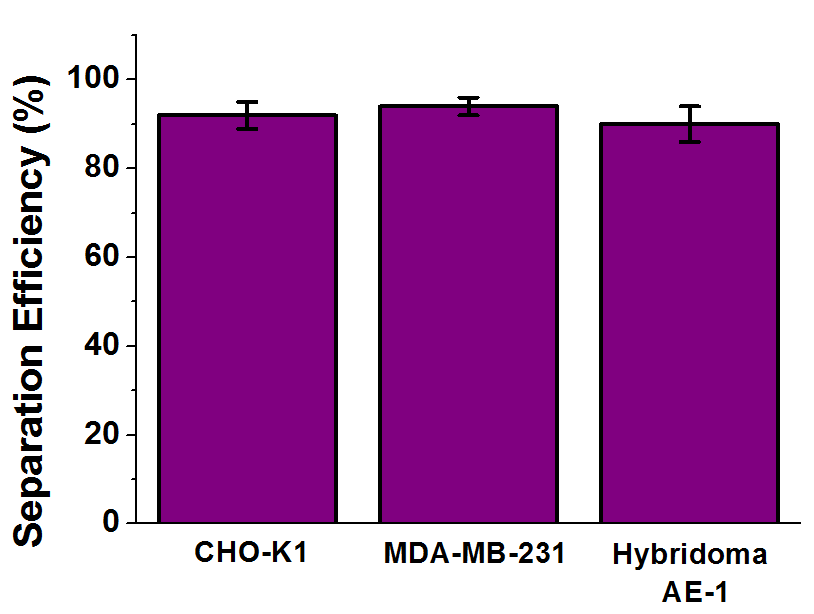** |
| --- |
| **Figure S1.** Recovery efficiency of inertial microfiltration system for processing of three different cell lines at concentration of 1×10^7^ cells/mL. |

| **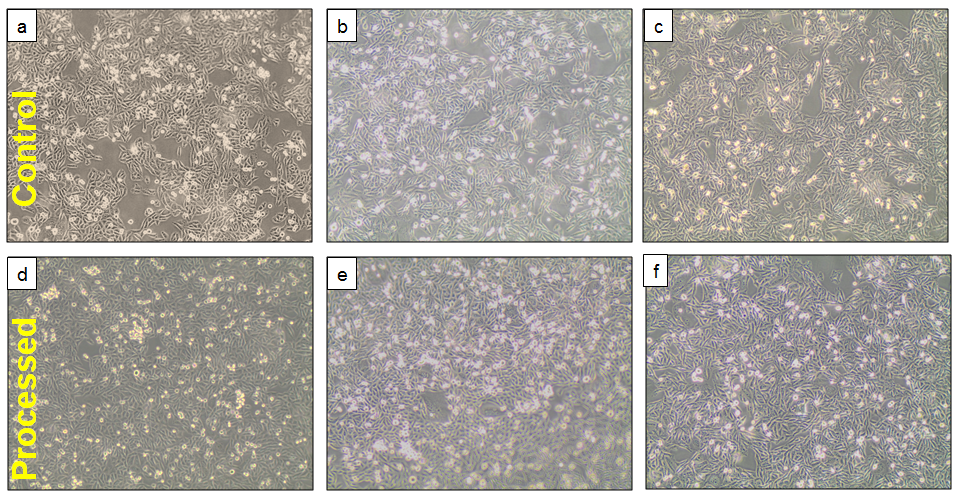** |
| --- |
| **Figure S2.** Phase contrast micrographs of cultures of control (unsorted) CHO cells (a-c) and sorted cells (d-f) by the inertial microfiltration system. The images indicate no significant differences between the morphology and proliferation rate of the cells suggesting high viability and sterility. |

| 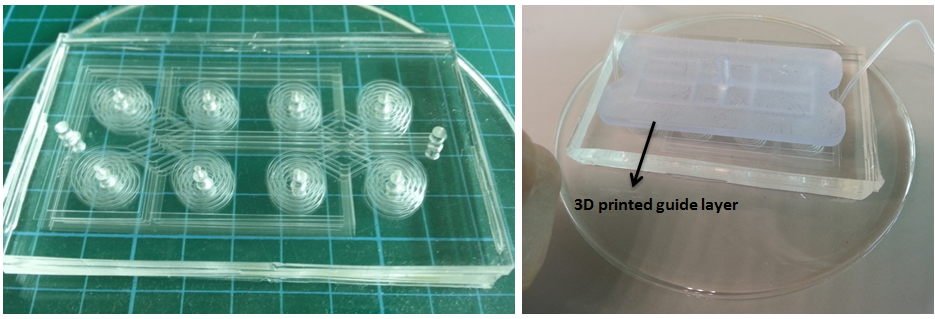 |
| --- |
| **Figure S3**. Optical image of a multiplexed inertial microfiltration system designed for separation of yeast cells. Similar to our system developed for mammalian cell retention, this system also consists of multiple layers of PDMS sheets with embossed microchannels bonded together for continues cell retention from large sample volumes. The microchannels used in this system has also trapezoidal cross-section with 30×70 µm channel dimensions. |

| **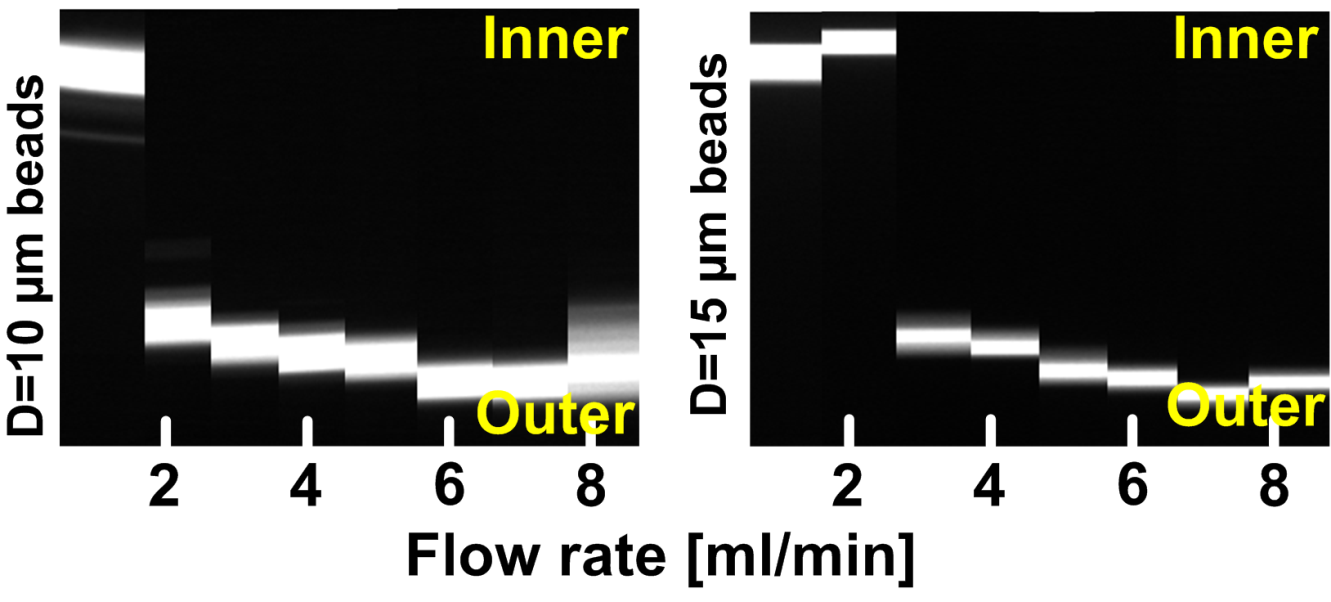** |
| --- |
| **Figure S4**. Top-view experimental observation of fluorescently microparticles at the outlet of a spiral device with trapezoidal cross-section (80×130 µm) used in this study for separation and fractionation of mammalian cells. In can be seen that at the flow rate of 6 mL/min, both 10 and 15 µm particles can be focused near the outer wall (i.e., filtration mode of our system) while at the 2 mL/min flow rate, the 10 µm beads are focused near the outer wall and 15 µm beads are focused near the inner wall (i.e., fractionation mode of our system). |
| **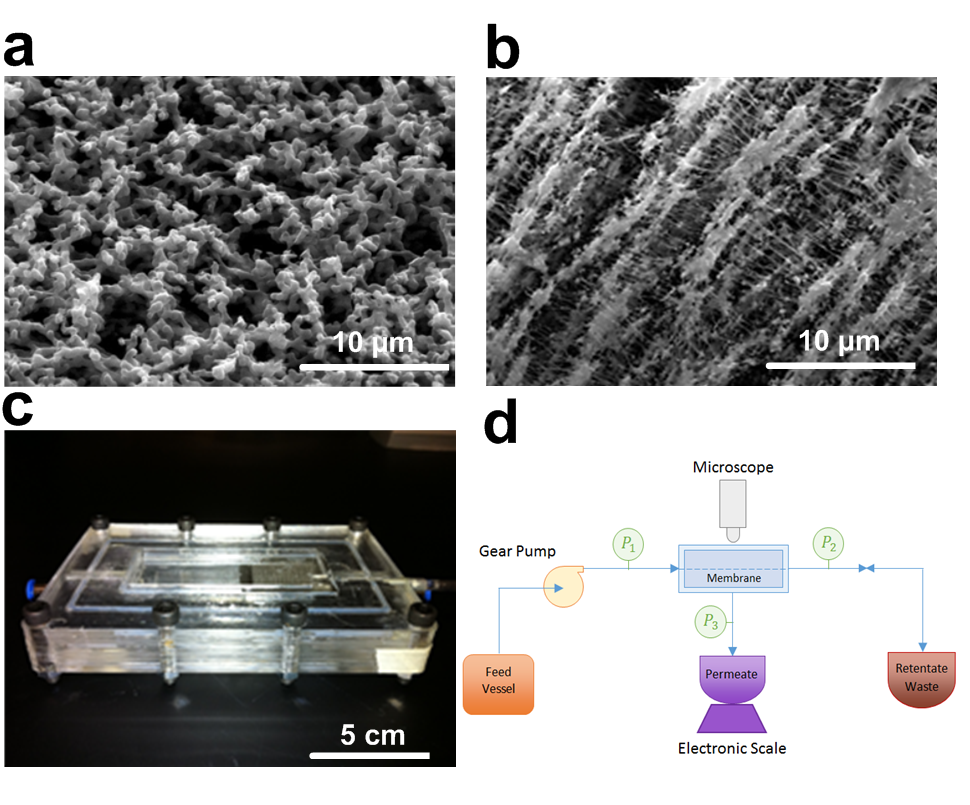** |
| **Figure S5**. (**a**) & (**b**) SEM photos of the Cellulose (Millipore, Cat. No. [AAWP04700](http://www.emdmillipore.com/SG/en/product/MF-Millipore-Membrane-Filter%2C-mixed-cellulose-esters%2C-Hydrophilic%2C-0.8 µm%2C-47 mm%2C-white%2C-plain,MM_NF-AAWP04700) ) and Teflon filter (Pall, Cat. No. P5PL047) with nominal pore size of around 1 µm used in this study for yeast separation. (**c**) Optical image of the cross-flow filtration setup made from PMMA and used for microfiltration tests. (**d**) Schematic representation of the experimental setup used for the tests. |

**SI Movie Legends**

**Movie S1**

High speed video (6400 fps) illustrating the separation of CHO cells at concentration of 10^6^ cell/mL using a single spiral channel at flow rate of 6 mL/min. Focused CHO cells (near the outer wall (bottom side)) are exiting the system from the outer outlet (i.e., can be returned to the cell culture flask or bioreactor) while clarified culture medium is collected continuously via the inner outlet.

**Movie S2**

High throughput cell separation (100 mL/min) using a multiplexed inertial microfiltration system (20 spiral channels).

**Movie S3**

High speed video (6400 fps) illustrating the separation of yeast cells at concentration of 0.1 gr/l using a single spiral channel at flow rate of 2 mL/min. Focused yeast cells (near the inner wall (bottom side)) are exiting the system from the outer outlet while clarified culture medium is collected continuously via the outer outlet. At this concentration, around 10-12% of yeast cells were escaping to the outer outlet (i.e., filtrate) during the 1^st^ cycle of enrichment; however, the filtrate can be further purified through 2^nd^ cycle of processing.

**Movie S4**

High speed video (6400 fps) illustrating the fractionation of CHO cells at concentration of 10^6^ cell/mL using a single spiral channel at flow rate of 1.5 mL/min. It can be seen that smaller CHO cells are trapped inside the Dean vortices and remained near the outer wall while bigger cells are focused near the inner wall, exiting the device from both outlets.
